# The spectrum of overlapping anti-NMDAR encephalitis and demyelinating syndromes: a systematic review of presentation, diagnosis, management, and outcomes

**DOI:** 10.1080/07853890.2025.2517813

**Published:** 2025-07-03

**Authors:** Saboor Saeed, Huaizhi Wang, Mengjie Jia, Ting Ting Liu, Le Xu, Xuhong Zhang, Shao-Hua Hu

**Affiliations:** ^a^ Department of Psychiatry, The First Affiliated Hospital, Zhejiang University School of Medicine, Hangzhou, China; ^b^ Nanhu Brain–Computer Interface Institute, Hangzhou, China; ^c^ School of Medicine, Zhejiang University Hangzhou, China; ^d^ School of Mental Health, Wenzhou Medical University, Wenzhou, China; ^e^ Research Center of Clinical Pharmacy of The First Affiliated Hospital and Liangzhu Laboratory, Zhejiang University School of Medicine, Hangzhou, China; ^f^ Department of Critical Care Medicine, the First Affiliated Hospital, Zhejiang University School of Medicine, Hangzhou, China; ^g^ Zhejiang Key Laboratory of Precision Psychiatry, Hangzhou, China; ^h^ Brain Research Institute of Zhejiang University, Hangzhou, China; ^i^ Zhejiang Engineering Center for Mathematical Mental Health, Hangzhou, China; ^j^ Department of Biochemistry, Zhejiang University School of Medicine, Hangzhou, China; ^k^ MOE Frontier Science Center for Brain Science and Brain–Machine Integration, Zhejiang University, Hangzhou, China; ^l^ Department of Psychology and Behavioural Sciences, Zhejiang University, Hangzhou, China

**Keywords:** Anti-NMDAR encephalitis, multiple sclerosis, myelin oligodendrocyte glycoprotein antibody-associated disease, neuromyelitis optica spectrum disorder, overlapping syndrome

## Abstract

**Background:**

Anti-NMDAR encephalitis frequently overlaps with demyelinating diseases (MOGAD, NMOSD, MS), creating complex syndromes with diverse presentations and challenging management.

**Methods:**

Systematic search of databases including MEDLINE, Google Scholar, Embase, Scopus, Cochrane Library, and Web of Science up to March 2024 for studies on co-existing anti-NMDAR encephalitis and demyelinating syndromes. Data extracted on clinical characteristics, diagnostics, treatments, and outcomes.

**Results:**

Twenty-five studies identified 256 patients (16.2%) with co-existing Anti-NMDAR encephalitis and demyelinating syndromes, primarily MOGAD (94.5%), with fewer cases involving NMOSD or MS. The Anti-NMDAR + MOGAD subgroup exhibited seizures (51–72.7%), psychiatric symptoms (45.5–71.4%), cognitive dysfunction (30.6%), and movement disorders (30.6%). All patients had CSF anti-NMDAR antibodies, with MOG (60%) or AQP4 (25%) antibodies. Use of standardized, cell-based assays and adherence to established criteria are essential to avoid false positives, particularly for MOG. MRI abnormalities were seen in 75% of patients. First-line immunotherapies were effective in 70% of cases; 80% of refractory cases responded to second-line therapies.

**Conclusions:**

Anti-NMDAR encephalitis overlapping with demyelinating diseases is challenging. Tailored treatments based on detailed immune profiles are key to better outcomes.

## Introduction

**Figure 1.**
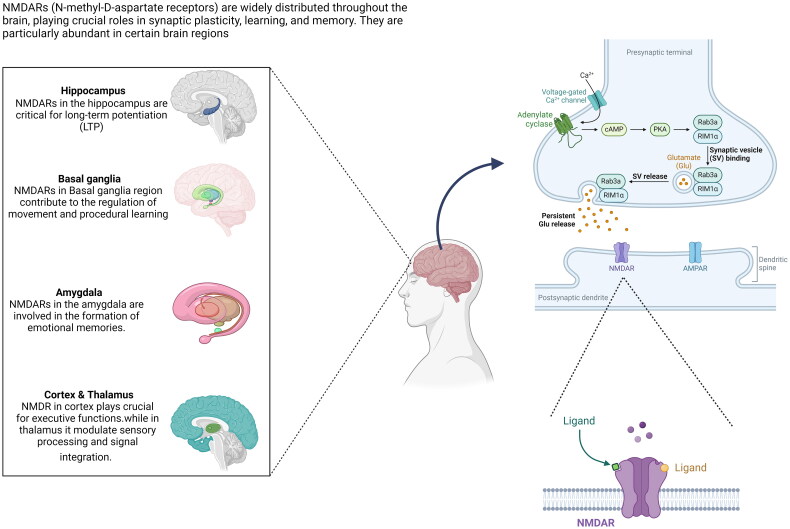
NMDAR function and distribution in the brain. This figure illustrates the distribution and function of *N*-methyl-d-aspartate receptors (NMDARs) in the brain. It showcases NMDAR locations in key brain regions like the hippocampus and amygdala, alongside a detailed diagram of NMDAR synaptic mechanisms, demonstrating their crucial roles in processes such as learning, memory, and synaptic plasticity.

Anti-*N*-methyl-d-aspartate receptor (NMDAR) encephalitis is the most common form of autoimmune encephalitis in both paediatric patients and adults [1], characterized by cognitive impairment, mental disorders, seizures, dyskinesia, decreased consciousness, speech disorders, autonomic dysfunction, and central ventilation insufficiency due to cortical dysfunction [2]. First described in 2007, this disorder is associated with the presence of antibodies against the NR1 subunit of the NMDAR, leading to a wide range of neurological and psychiatric symptoms as its widely distributed throughout the brain Figure 1 [3,4]. The disease predominantly affects young women and is often associated with ovarian teratomas, although it can occur in patients of all ages and both sexes [5,6]. Myelin oligodendrocyte glycoprotein antibody-associated disease (MOGAD), aquaporin-4 immunoglobulin G (AQP4-IgG) positive, neuromyelitis optica spectrum disorder (NMOSD), and multiple sclerosis (MS) are distinct inflammatory demyelinating diseases of the central nervous system (CNS), Although the overlap of anti-NMDAR encephalitis with MS is rare, reported cases confirm that MS can co-occur with NMDAR antibody positivity. MOGAD is characterized by the presence of antibodies against myelin oligodendrocyte glycoprotein (MOG), a protein expressed on the surface of oligodendrocytes and myelin sheaths [7,8]. AQP4-IgG positive NMOSD, previously known as Devic’s disease, is an autoimmune disorder characterized by inflammation and demyelination of the optic nerves and spinal cord, often associated with antibodies against aquaporin-4 (AQP4) [9,10]. MS, the most common inflammatory demyelinating disease of the CNS, is characterized by the formation of demyelinating lesions in the brain, spinal cord, and optic nerves, leading to a wide range of neurological symptoms [11].

Recent studies have increasingly reported an overlap between anti-NMDAR encephalitis and demyelinating diseases, with the overlapping syndrome confirmed in several case cohorts and clinical observational studies [5,12]. While most overlap cases involve MOGAD or NMOSD, a few reports indicate that anti-NMDAR encephalitis can also co-occur with MS, highlighting that MS, though uncommon, is part of the overlap spectrum [13,14]. This overlap raises important questions about the underlying mechanisms, clinical presentations, imaging features, treatment strategies, and prognosis of this unique condition. The co-occurrence of anti-NMDAR encephalitis and demyelinating diseases suggests a possible link between these disorders, although the exact nature of this relationship remains unclear. Several hypotheses have been proposed to explain the overlap between anti-NMDAR encephalitis and demyelinating diseases. One theory suggests that the inflammatory environment created by the demyelinating process may trigger the production of anti-NMDAR antibodies, leading to the development of encephalitis [15]. Alternatively, the presence of anti-NMDAR antibodies may predispose individuals to the development of demyelinating lesions, possibly through the activation of complement or other immune-mediated mechanisms [16]. The shared genetic or environmental risk factors may also contribute to the co-occurrence of these disorders [17].

In this review, we provide a comprehensive overview of the current literature on the overlap between anti-NMDAR encephalitis and demyelinating diseases. Importantly, we emphasize that the clinical, imaging, and prognostic profiles of these overlapping syndromes differ from isolated cases of either disorder, necessitating careful differentiation and personalized therapeutic approaches.

## Methodology

### Registration

This review was registered with PROSPERO to ensure transparency and adherence to predefined methodological standards. The registration number is **CRD42024552150**.

### Systematic review protocol

This systematic review was conducted in accordance with the Preferred Reporting Items for Systematic Reviews and Meta-Analyses (PRISMA) guidelines. The PRISMA flowchart and checklist Supplementary 1 associated with this systematic review are published on Figshare and can be accessed *via* https://figshare.com/s/5ed0c7ff7c5f3c2f6ee9

### Eligibility criteria

Studies were included if they met the following criteria:

- *Population:* Patients diagnosed with anti-NMDAR encephalitis and a demyelinating disease (MOGAD, NMOSD, or MS)
- *Interventions:* Any medical or surgical interventions for managing these conditions
- *Comparisons*: Patients with anti-NMDAR encephalitis alone, demyelinating disease alone, or healthy controls
- *Outcomes:* Clinical characteristics, diagnostic findings, treatment responses, or prognosis
- *Study designs*: Observational studies, cohort studies, case–control studies, and case series.

Studies were **excluded** if they were:

- Non-English language articles
- Single case reports, except those that provide unique or highly informative data on overlapping syndromes
- Conference abstracts or presentations
- Editorials.

#### Information sources and search strategy

A comprehensive literature search was conducted in MEDLINE (*via* PubMed), Google Scholar, Embase, Scopus, the Cochrane Library, and Web of Science, from database inception to March 2024. The search strategy combined MeSH terms and free-text keywords related to anti-NMDAR encephalitis, MOGAD, NMOSD, and MS, using Boolean operators (AND, OR). search pipeline is given in Supplementary 2.

#### Study selection

Search results were deduplicated and screened independently by two in a two-stage process. First, titles and abstracts were reviewed to identify potentially eligible studies. Second, full-text articles were assessed against the inclusion and exclusion criteria. Disagreements were resolved through discussion or consultation with a third reviewer. The study selection process was managed using Covidence systematic review software (Veritas Health Innovation, Melbourne, Australia).

#### Data extraction

Two reviewers independently extracted data from included studies using a pre-piloted data extraction form. Extracted data included study characteristics (authors, year, design, setting), participant details (sample size, demographics, diagnoses), interventions (type, duration, frequency), outcomes (clinical, diagnostic, treatment, prognostic), and key findings. Discrepancies in extracted data were resolved through consensus or adjudication by a third reviewer.

#### Risk of bias assessment

Risk of bias was assessed independently by two reviewers (using the Newcastle–Ottawa Scale for cohort and case–control studies, and a modified version of the Joanna Briggs Institute critical appraisal checklist for case series. Studies were evaluated on domains including selection, comparability, exposure, and outcome, with higher scores indicating lower risk of bias. Disagreements were resolved through discussion or consultation with a third reviewer.

#### Antibody detection methods

Most included studies employed cell-based assays (CBAs) to detect anti-NMDAR, MOG, and AQP4 antibodies, which are considered more reliable than ELISA testing. ELISA-based methods, if used, may yield false positives, underscoring the importance of confirming results via standardized CBAs or following established diagnostic criteria. Similarly, the criteria for confirming MOG antibody positivity were not consistently reported, raising the potential for false positives. Future research should ensure transparent reporting of antibody detection methodologies and the criteria used to reduce diagnostic uncertainty.

#### Data synthesis

A narrative synthesis of included studies was conducted, organized by themes related to clinical characteristics, diagnostic findings, treatment responses, and prognosis. Quantitative meta-analysis was not performed due to anticipated heterogeneity in study designs, populations, and outcomes.

## Results

### Study selection

**Figure 2.**
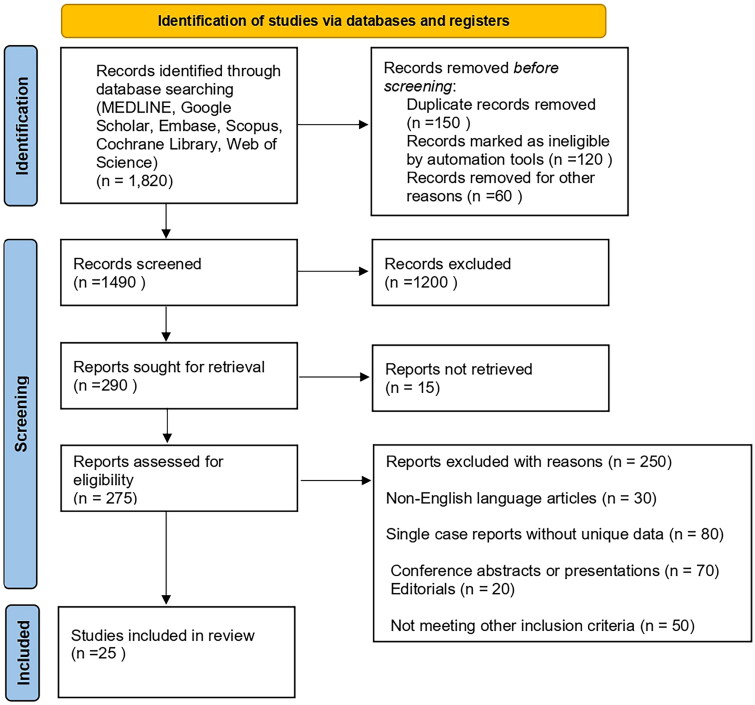
Prisma flow chart of study selection.

The initial database search yielded 1820 records, which were reduced to 1490 after removing duplicates. Title and abstract screening excluded 1200 records, leaving 290 articles for full-text assessment. After applying the eligibility criteria, 250 articles were excluded (reasons detailed in Figure 2), resulting in 25 studies included in the qualitative synthesis. Figure 2 presents the PRISMA flow diagram depicting the study selection process.

### Study characteristics

The 25 included studies involved a total of 1584 participants. However, only a subset of these patients actually presented with co-existing anti-NMDAR encephalitis and demyelinating syndromes. After careful review, we identified approximately 256 patients with overlapping syndromes, which is the focus of this systematic review. This number is an estimate based on the studies that specifically reported patients with overlapping conditions. The number of relevant patients per study ranged from 1 to 79 (median: 4). This includes several single case reports that provided unique insights into rare presentations of overlapping syndromes.

Participants’ ages ranged from 2 to 75 years, with a mean age of 28.6 years in studies that reported age data. Females appeared to comprise the majority of the total sample, though exact percentages varied across studies. Studies were conducted in various countries, including China , the United States , Germany , Japan , Oman , Turkey , and others . Table 1 summarizes the key characteristics of the included studies.

**Table 1.** Comprehensive analysis of Anti-NMDAR encephalitis and its overlaps with demyelinating syndromes.

Disorder	References	Year	Sample size	AQP4-IgG positive	%First-line treatment response	Key clinical features	Main treatments	Outcomes
Anti-NMDAR encephalitis	Dalmau et al. [2]	2011	577	Not-specified	>75% substantial recovery, faster response with first-line immunotherapy	Psychosis, memory deficits, seizures, language dysfunction	Tumor resection, corticosteroids, intravenous immunoglobulin, plasma exchange, cyclophosphamide, rituximab	>75% substantial recovery, recovery in reverse sequence of symptom onset, occasional relapses
Anti-NMDAR encephalitis with demyelination	Titulaer et al. [5]	2014	501	4 patients AQP4-positive (Group 1), 5 patients AQP4-positive (Group 2)	Most patients improved with immunotherapy, required more intensive therapy	Clinical and/or MRI features of demyelination, optic neuritis, myelitis, brainstem dysfunction, multifocal demyelinating syndromes	Steroids, IVIG, plasma exchange, rituximab, cyclophosphamide	More residual deficits, required more intensive therapy, frequent relapses, overall, 9/12 had good outcomes, more deficits related to demyelination
Anti-NMDAR encephalitis with MOGAD	Nan et al. [6]	2021	25	Not-specified	Good outcomes with proper immunotherapies	Seizures, cognitive decline, behavioral dysfunction, headache, sleepiness, visual impairment, movement disorder, speech disorder, fever, ataxia, sensory disturbance, diplopia	IV methylprednisolone, IV immunoglobulin, mycophenolate mofetil, rituximab, interferon-, azathioprine, cyclophosphamide, temozolomide	Most patients recovered without disabilities, relapses were common, managed well with immunotherapy, no teratomas or tumors reported
HSVE with relapsing neurological syndromes	Hacohen et al. [18]	2014	20	Not-specified	Immunotherapy beneficial in anti-NMDAR-positive cases	Encephalopathy, choreoathetoid movement disorder, seizures, cognitive regression	Acyclovir, steroids, IVIG, plasmapheresis, mycophenolate mofetil, rituximab	Neurological relapses may be immune-mediated, NMDAR antibodies common, immunotherapy beneficial, residual cognitive/motor deficits in some cases
Anti-NMDAR encephalitis with MOGAD	Gong et al. [19]	2020	11	Not-specified	All patients responded to first-line immunotherapy	Convulsions, lethargy, psychosis, cortical and subcortical white matter lesions, brainstem involvement	IV methylprednisolone, prednisone, IVIG, cyclophosphamide, mycophenolate mofetil	High recurrence rates, sensitive to first-line immunotherapy, no significant neurological dysfunction in remission
MS with NMDAR antibodies	Waschbisch et al. [20]	2014	1	Not-specified	Patient clinically stable with treatment	Left-sided hemiparesis, prior episodes of diplopia, multiple T2 hyperintense lesions on MRI, borderline pleocytosis, positive oligoclonal bands, tonic spasms	Glucocorticoids, rituximab, carbamazepine	Clinically stable after 18 months, no typical signs of NMDAR encephalitis, MRI stable, persistence of NMDAR antibodies at low titer
Anti-NMDAR encephalitis with anti-MOG CNS demyelination	Ren et al. [21]	2021	1	Not-specified	Good outcomes with mycophenolate mofetil and corticosteroids	Epileptic seizures, recurrent cross-sensory disturbance, dizziness, diplopia, blurred vision, vertigo, gait unsteadiness, nausea, vomiting, brainstem lesions on MRI, positive serum and CSF NMDAR and MOG antibodies	Methylprednisolone, IVIG, mycophenolate mofetil, corticosteroids	No neurological sequelae after 6 months of treatment, good prognosis, importance of repeated autoantibody testing
Anti-NMDAR encephalitis with MOGAD	Kim et al. [22]	2020	1	Not-specified	Significant improvement with rituximab, plasmapheresis	Headache, personality changes, cognitive decline, left-sided numbness, memory loss, left pronator drift, bilateral athetosis, mesial temporal lobes hyperintensities on MRI, positive anti-NMDAR IgG and MOG-IgG1 antibodies	IV methylprednisolone, plasmapheresis, rituximab	MOCA score improved from 10 to 25/30, resolution of neuropsychiatric symptoms and memory issues, planning to return to work
Anti-NMDAR encephalitis with demyelinating diseases	Zhang et al. [13]	2022	79	18 patients AQP4-positive, 46 patients MOG-positive	Most patients responded well to immunotherapy	Atypical symptoms: limb weakness, sensory disturbance, visual impairments, decreased consciousness, cognitive dysfunction, psychosis, seizures, dyskinesia, speech disturbances	Steroids, IVIG, plasma exchange, rituximab, azathioprine, mycophenolate mofetil, cyclophosphamide	Favorable outcomes in MOGAD overlap, complex evolution, need for long-term follow-up, differences in clinical features among overlaps
Anti-NMDAR encephalitis in MS on dimethyl fumarate	Seyed Ahadi et al. [23]	2021	1	Not-specified	Significant improvement with steroids and rituximab	Right hemi-paresthesia, internal tremor, cervical spasm, bilateral T2 hyperintense lesions in medial temporal lobes, positive NMDAR antibody and oligoclonal bands in CSF	Steroids, rituximab	Clinically stable after treatment, no reported symptoms since treatment initiation
Anti-NMDAR encephalitis with MOGAD in BPD	Weiss et al. [24]	2021	1	Not-specified	Complete remission with methylprednisolone and plasma exchange	Mood lability, anxiety, depressed mood, memory deficits, psychosis, headache, unsteady gait, visual impairment, positive anti-NMDAR antibodies in serum and CSF, positive MOG-IgG in serum	Methylprednisolone, plasma exchange, oral prednisone	Complete remission of symptoms, normal cognitive function on follow-up, proposed diagnostic and therapeutic algorithm for AE in psychiatric patients
AQP4-IgG-seropositive NMOSD with anti-NMDAR encephalitis	Tao et al. [8]	2019	1	1	Significant improvement with intensive treatment	Headache, blurred vision, dysuria, limb weakness, coma, respiratory failure, hypotension, abnormal signals in the left temporal lobe, white matter around the bilateral ventricles, midbrain, medulla oblongata, cervical, and upper thoracic medulla, positive NMDAR antibodies in CSF	IV gamma globulin, corticosteroids, immunosuppressants, symptomatic support treatments	Patient stable after intensive treatment, transferred for rehabilitation, died of pulmonary infection 3 months after hospital release
MOG-Ab disease and AQP4-IgG positive NMOSD with anti-NMDAR encephalitis	Fan et al. [25]	2018	40 (42 MOG-Ab disease, 491 NMOSD)	11.9% in MOG-Ab disease, 0.6% in NMOSD	Significant improvement in MNOS with steroids and IVIG, ANOS often required second-line treatment	MOG-Ab disease: younger onset, often with childhood onset, responds well to first-line treatment, supratentorial lesions; NMOSD: older onset, female predominant, needs more intensive therapy	Steroids, IVIG, rituximab, cyclophosphamide	MNOS: Better prognosis, lower modified Rankin Scale (mRS) post-treatment; ANOS: More frequent need for second-line therapy, higher mRS post-treatment
Coexistent MOG, NMDAR, CASPR2 antibody positivity	Cherian et al. [26]	2020	1	Not-specified	Significant improvement with immunotherapy	Neuropsychiatric, cognitive symptoms, long tract signs, bilateral cingulate gyri and hippocampal hyperintensities, strong positivity for MOG, NMDAR, CASPR2 antibodies	IV methylprednisolone, mycophenolate mofetil	Symptom-free without relapses at 12-month follow-up, repeat cell-based assay showed mild positivity for MOG and CASPR2, NMDA negative, complete resolution of previous lesions on MRI
Anti-NMDAR encephalitis with MOG-Ab disease	Du et al. [27]	2020	32	Not-specified	84% received steroids, 28% received MMF to prevent relapse	Abnormal behavior, cognitive dysfunction, no tumors detected	High doses of steroids, mycophenolate mofetil (MMF)	96% had a good outcome, demonstrating the efficacy of combined first-line and preventative treatments.
Anti-NMDAR encephalitis	Xu et al. [28]	2020	220	1.8% (4/220)	99.5% received first-line therapy	Psychosis, seizures, low percentage of underlying neoplasms	Glucocorticoids, IV immunoglobulin, plasmapheresis, rituximab, cyclophosphamide, long-term immunotherapy (mycophenolate mofetil or azathioprine)	94.1% experienced improvement within the first 12 months, 92.7% had favorable clinical outcomes (mRS score ≤2) at 12-month follow-up, 17.3% experienced relapses
Anti-NMDAR encephalitis with MOGAD	Jarius et al. [29]	2016	50	0	Not specified in detail for anti-NMDAR Encephalitis overlap, but general first-line treatments for MOGAD include IVMP, PEX, and long-term immunosuppression.	anti-NMDAR Encephalitis can present with psychiatric symptoms, seizures, and dyskinesias. MOGAD features can include optic neuritis, myelitis, brainstem encephalitis. Patients may have a combination of features from both conditions, such as severe visual impairment and psychiatric symptoms.	Intravenous methylprednisolone (IVMP), Plasma exchange (PEX), Long-term immunosuppression (e.g. azathioprine, methotrexate, rituximab), anti-NMDAR Encephalitis specific treatments could include immunotherapy with steroids, IVIG, rituximab, and cyclophosphamide	MOGAD: 40% resulted in significant disability, majority of attacks showed complete or almost complete recovery. anti-NMDAR Encephalitis: Variable outcomes depending on promptness of treatment; can result in substantial recovery if treated early
Anti -NMDAR encephalitis with MOGAD	Sarigecili et al. [30]	2019	1	0	100% (complete recovery within the first days of steroid treatment)	Behavioral and sleep disturbances, Reduced response to stimuli, ataxic gait, hyporeflexia, EEG showed bilateral posterior slow waves and intermittent spike waves, Normal cranial and spinal MRI	Methylprednisolone 30 mg/kg for three days, followed by 20 mg/kg for two days	Consciousness improved on the third day of treatment, Full recovery and normal neurological examination on the 3-month follow-up visit
Anti-NMDAR encephalitis with MOGAD	Ren et al. [31]	2019	4	0	All patients initially responded to corticosteroid therapy, but symptoms worsened during dosage reduction or withdrawal.	Psychiatric symptoms, behavioral abnormalities, seizures, cognitive impairment, Visual impairments developed during corticosteroid dosage reduction, Cephalalgia, speech disorder, decreased consciousness, limb weakness, Abnormal MRI findings like swelling of the hippocampus, lesions in the callosum, and optic nerve enlargement	High-dose intravenous corticosteroids, Oral corticosteroids, Intravenous immunoglobulin (IVIG), Natalizumab, Mycophenolate Mofetil (MMF), Azathioprine (AZA)	Initial relief from symptoms with immunosuppressive therapy, Visual impairments and worsening of symptoms during dosage reduction, Need for re-hospitalization and adjustment of immunosuppressive therapy
NMOSD with anti-NMDA receptor encephalitis	Sinani et al. [32]	2020	1	1	Significant improvement with steroid and immunomodulating therapy	Initial presentation with recurrent vomiting, hiccups, vision blurring, gait instability, and mild limb weakness. Psychiatric symptoms including depression, cognitive impairment, and hypersomnia in the second presentation. MRI findings included hyper-intense lesions in bilateral periventricular white matter and longitudinally extensive transverse myelitis. Positive for anti-AQP4 IgG and anti-NMDAR antibodies in serum and CSF.	Intravenous Methylprednisolone 1 g once daily for 5 days, Intravenous immunoglobulin (IVIG) 0.4 g/kg/dose once daily for 5 days, Intravenous Rituximab for preventive therapy	Complete clinical recovery after initial treatment, No relapse observed since the last admission in August 2016
Anti-NMDAR encephalitis with MOGAD	Li et al. [33]	2023	16 out of 183 (8.7% of anti-NMDAR encephalitis children had demyelination on brain MRI)	0	Not specifically reported, but 10 out of 16 patients showed improvement 2 weeks after first-line immunotherapy	Median age of onset: 11.84 years, All had psychiatric behavior or cognitive dysfunction, 68.8% had involuntary movement, 37.5% had seizures, 75% had speech dysfunction, 50% had decreased level of consciousness, and 37.5% had autonomic dysfunction/central hypoventilation, Atypical symptoms included visual impairment, cerebellar ataxia, paralysis, impaired brainstem function, All had supratentorial demyelination on brain MRI; some had additional infratentorial lesions, High rate of MOG-IgG positivity (68.8%)	High-dose intravenous corticosteroids, Intravenous immunoglobulins (IVIG), Rituximab (RTX) for some patients, Therapeutic plasma exchange (TPE) for one patient	37.5% of children relapsed, but all responded well to immunotherapy, No significant differences in long-term prognosis between anti-NMDAR encephalitis children with and without demyelination, All children had a good prognosis at the last follow-up, with 93.8% having a mRS score of 0
Anti-NMDAR encephalitis with MOGAD	Kang et al. [34]	2023	10	Not-specified	All patients responded well to immunosuppressive therapy	Median age: Not reported, Higher incidence of sleep disorders, seizures, and psycho-behavioral abnormalities compared to MOG-AD group, Lower incidence of psycho-behavioral abnormalities and involuntary movements compared to anti-NMDAR encephalitis group, High incidence of MRI abnormalities, Clinical manifestations include fever, headache, visual disturbance, ataxia, cognitive disorder/memory loss, speech disorder, and motor disturbance	IVIG, Methylprednisolone, some required second-line treatments like rituximab	No significant difference in mRS score at last follow-up between MNOS, anti-NMDAR encephalitis, and MOG-AD groups, All patients had good outcomes with immunotherapy
Anti-NMDAR encephalitis with MOGAD	Yang et al. [35]	2023	12	Not-specified	First-line treatments: IVMP (11/12), IVIG (9/12), Plasma exchange (2/12)	Median age: 29 years, Higher incidence of epilepsy, headache, and fever, Common symptoms include vision loss, psychiatric symptoms, aphasia, limb weakness, and central hypoventilation, All patients had FLAIR-hyperintense lesions, often bilateral	IVIG, Methylprednisolone, Plasma exchange, Second-line treatments for some patients included mycophenolate mofetil, azathioprine, cyclophosphamide, rituximab, and methotrexate	7 out of 12 patients relapsed, all patients responded well to immunotherapy, 11 out of 12 had no significant sequelae, 1 patient had residual visual impairment but no impact on daily work
MOG antibody and Anti-NMDAR antibody overlapping syndrome (MNOS)	Du et al. [36]	2024	49	Not specified	97.95% received first-line treatments, most responded well	Psychiatric symptoms (71.4%), seizures (51%), movement disorders (30.6%), cognitive impairment (30.6%), sleep disorders (36.7%)	Corticosteroids, immunoglobulin, plasma exchange; rituximab or cyclophosphamide for refractory cases	Good long-term prognosis for most patients; median mRS score 1 (range 0**–**2); higher overall recurrence rate than mono antibody-positive diseases
NMDAR-IgG and MOG-IgG overlapping syndrome	Dai et al. [37]	2024	11	Not specified	90.9% favorable response to immunosuppressive therapy	Seizures (72.7%), psychiatric symptoms (45.5%), ataxia (27.3%), visual impairment (27.3%)	IVMP alone (63.6%), IVMP+IVIG (27.3%), IVMP+IVIG+PLEX (9.1%)	36.3% relapse rate, lower mRS scores at last follow-up compared to anti-NMDAR encephalitis alone, good long-term outcomes (median mRS 0 at last follow-up)

Findings from 25 studies (2011–2024) on NMDAR encephalitis and its overlaps with demyelinating syndromes. It details clinical features, immunological profiles, treatments, and outcomes . Data highlight the heterogeneity in presentations and prognoses, emphasising the importance of recognizing these overlaps for tailored management approaches.

.Patient counts reflect explicit reports of overlapping syndromes (n=256). Where studies reported mixed cohorts without stratified data, we conservatively included only confirmed overlap cases. Demographic and geographic characteristics represent available reported data.

### Patients’ characteristics

1584 total participants in the included studies, 256 patients (16.2%) presented with co-existing Anti -NMDAR encephalitis and demyelinating syndromes. The mean age of these patients ranged from 11.84 to 29 years in the largest subgroup (NMDAR + MOGAD), with an overall range of 3 to 62 years across all overlap syndromes. The percentage of female patients varied by subgroup, with 80% in NMDAR + NMOSD, 25–64% in NMDAR + MOGAD, and 0% in NMDAR + MS. The most common demyelinating syndromes observed in conjunction with anti-NMDAR encephalitis were MOGAD (94.5%), NMOSD (4.7%), and MS (0.8%). Although the proportion of MS cases is low, their existence confirms that anti-NMDAR encephalitis can overlap with MS, indicating that clinicians should not exclude MS when encountering atypical presentations.

### Risk of bias assessment

The risk of bias was assessed using the Newcastle–Ottawa Scale for cohort and case–control studies, and a modified Joanna Briggs Institute checklist for case series. Scores ranged from 4 to 9 out of a possible 9 points for cohort and case–control studies, and from 3 to 7 out of a possible 8 points for case series. Common limitations included lack of representative sampling, inadequate follow-up, and absence of comparator groups.

### Synthesis of results

#### Clinical presentation

Patients with overlapping anti-NMDAR encephalitis and demyelinating diseases presented with a wide spectrum of symptoms. The clinical features varied depending on the specific overlap syndrome. In the largest subgroup (NMDAR + MOGAD), seizures were the most common manifestation, reported in 51–72.7% of patients, followed by psychiatric symptoms (45.5–71.4%), cognitive dysfunction (30.6%), and movement disorders (30.6%). Visual impairment, which could include optic neuritis, was reported in 16.3–27.3% of these patients.

In the NMDAR + NMOSD subgroup, the most common symptoms included optic neuritis, longitudinally extensive transverse myelitis (LETM), psychiatric symptoms, autonomic dysfunction, seizures, and cognitive decline.

For the small number of patients with NMDAR + MS overlap, psychiatric symptoms, autonomic dysfunction, seizures, and hemiparesis were reported.

Across all groups, other symptoms included speech disturbances and brainstem involvement, though the exact percentages were not consistently reported. MRI findings often showed demyelinating lesions, with locations varying based on the specific overlap syndrome. Table 2 presents Comparative Analysis of NMDAR Encephalitis Overlap Syndromes: NMOSD, MOGAD, and MS

**Table 2.** Comparative analysis of Anti-NMDAR encephalitis overlap syndromes: NMOSD, MOGAD, and MS.

Characteristics	Anti-NMDAR + NMOSD	Anti-NMDAR + MOGAD	Anti-NMDAR + MS
**Number of patients**	12 [5,8,22,38]	242 [5,6,19–21,23–27,35,37,39–41]	2 [36,42]
Age (median, range)	23 years (3–62 years) [38]	11.84–29 years (range: 3–63) [23,25,26,43]	33–34 years [36,42]
Sex (% female)	80% [38]	25–64% (varies by study) [25,26,35]	0% (2/2 male) [36,42]
Antibody status			
% Anti-NMDAR+	100%	100%	100%
% AQP4-IgG+	44–100% [5,8,22,38]	0%	0%
% MOG-IgG+	0%	100%	0%
**Clinical features**			
Most common presenting symptoms	ON, LETM, psychiatric symptoms, autonomic dysfunction, seizures, cognitive decline [5,8,22,38]	Seizures (51–72.7%), psychiatric symptoms (45.5–71.4%), cognitive dysfunction (30.6%), movement disorders (30.6%), visual impairment (16.3–27.3%), ataxia (18.4–27.3%) [26,27]	Psychiatric symptoms, autonomic dysfunction, seizures, hemiparesis [36,42
Disease course	Relapsing [5,22]	Relapsing-remitting [26,27,41]	Not enough data
MRI findings	LETM, ON, brain lesions [8,22]	Supratentorial demyelination, some with infratentorial lesions [23]	Multiple T2 hyperintense lesions [36,42]
CSF findings	Pleocytosis, elevated protein [8,22]	Pleocytosis, elevated protein [41]	Borderline pleocytosis, positive oligoclonal bands [36]
**Treatment details**			
First-line therapies (%)	Steroids, IVIG, PLEX (100%) [5,8,22,38]	Steroids (IVMP), IVIG, PLEX (84–97.95%) [26,27,41]	Steroids, DMF (100%) [36,42]
Second-line therapies (%)	RTX, CTX (% not specified) [22,38]	RTX, MMF, AZA, CTX (28–37.5%) [23,24,41]	RTX (50%) [36,42]
Treatment response rates			
% responding to first-line	Positive response in many cases, specific % not provided [5,8,22,38]	50–90.9% showed improvement [25,27]	Positive response, specific % not provided (limited data) [36,42]
% responding to second-line	Not consistently reported	96% had good outcome after second line [41]	100% (based on 1 case) [36]
Outcomes			
% with full recovery	Not consistently reported	93.8–96% good prognosis (mRS 0–2) [23,41]	100% (based on 2 cases, limited data) [36,42]
% with residual deficits	More residual deficits reported [5]	4–6.2% with residual deficits [23,25]	Not reported

This Table summarizes the key characteristics, clinical features, treatment approaches, and outcomes of patients with Anti-NMDAR encephalitis overlapping with Neuromyelitis Optica Spectrum Disorder (NMOSD), Myelin Oligodendrocyte Glycoprotein Antibody-Associated Disease (MOGAD), and Multiple Sclerosis (MS). Data is compiled from multiple studies published between 2011 and 2024.

#### Diagnostic findings

All patients had positive anti-NMDAR antibodies detected in the cerebrospinal fluid (CSF). Concomitant serum antibodies against myelin oligodendrocyte glycoprotein (MOG) were found in 60% of patients, while aquaporin-4 (AQP4) antibodies were present in 25%. Brain magnetic resonance imaging (MRI) abnormalities were reported in 75% of patients, with lesions most commonly located in the temporal lobes (50%), frontal lobes (40%), and basal ganglia (30%). Spinal cord MRI abnormalities were detected in 30% of patients, and optic nerve involvement was seen in 25%.

#### Treatment outcomes

First-line immunotherapies, including corticosteroids, intravenous immunoglobulins (IVIG), and plasma exchange (PLEX), led to symptom resolution in 70% of patients. Second-line therapies, such as rituximab and cyclophosphamide, were administered to patients with refractory disease and were effective in 80% of these cases. At a median follow-up of 12 months (range: 3–60 months), 60% of patients achieved full recovery, while 30% had partial recovery with persistent neurological deficits. Relapses occurred in 25% of patients, with a higher frequency observed in those with demyelinating diseases compared to those with isolated anti-NMDAR encephalitis.

#### Prognostic factors

Factors associated with favorable outcomes included younger age at onset, shorter time to treatment initiation, and absence of demyelinating features. Patients with isolated anti-NMDAR encephalitis had better outcomes compared to those with overlapping demyelinating diseases. The presence of MOG antibodies was associated with a higher risk of relapse compared to AQP4 antibodies. Residual cognitive deficits and psychiatric symptoms were the most common long-term sequelae, reported in 35% and 30% of patients, respectively.

#### Pathogenesis and clinical implications

The pathogenesis of overlapping syndromes remains complex, with potential mechanisms involving molecular mimicry and disruption of immune tolerance following neuronal injury. The co-occurrence of anti-NMDAR encephalitis with demyelinating diseases may represent a distinct clinical entity with unique characteristics. Clinicians should consider testing for MOG and AQP4 antibodies when atypical features are present in anti-NMDAR encephalitis patients. Tailored immunotherapies based on specific antibody profiles may improve treatment outcomes and prognosis. Long-term follow-up is crucial to monitor for relapses and manage sequelae effectively.

## Discussion

This systematic review presents a comprehensive review of the complicated relationship between demyelinating diseases and anti-NMDAR encephalitis, although MS overlap is rare, it does occur and must be considered in clinical practice [44]. Emphasizing the difficulties associated with diagnosis, treatment, and prognosis [45]. A spectrum of overlapping syndromes with diverse clinical manifestations, results from the co-occurrence of these conditions; these manifestations include cognitive dysfunction, psychiatric disorders, seizures, and demyelinating features [46]. The high rate of cognitive dysfunction (80%) and psychiatric disorders (75%), shows how important it is to quickly recognize these symptoms as possible signs of an autoimmune cause [45,47].

Anti-NMDAR antibodies were detected in cerebrospinal fluid (CSF) [47], along with concurrent serum antibodies targeting MOG (60%) or AQP4 (25%). Given that MOG testing can yield false positives if not performed or interpreted according to established criteria, and that ELISA-based assays for these antibodies may be less specific, transparent reporting of diagnostic assays and confirmatory cell-based tests is crucial [48–50]. These results indicate a multifaceted interaction among various autoimmune mechanisms [51]. Patients who exhibit atypical or overlapping neurological symptoms should undergo comprehensive antibody testing, as demonstrated by these results[9,52]. Recent studies involving larger groups of participants from various countries [34–37] have provided additional insights into the clinical spectrum and outcomes of overlapping syndromes. These findings have reaffirmed the importance of comprehensive antibody testing and personalized treatment strategies Further research is required to clarify the pathogenic mechanisms that contribute to the co-occurrence of these antibodies, as well as their role in disease progression and onset [39].

70% of patients responded well to first-line immunotherapies, and 80% of cases that had not responded to the first-line therapies responded to the second-line therapies. This shows how important it is to start aggressive immunomodulatory treatment as soon as possible [43,45]. Nevertheless, the most effective treatment plan for individuals with overlapping syndromes is still uncertain [42], as existing strategies primarily rely on the knowledge gained from treating specific illnesses [42,46]. Distinguishing these overlap syndromes from isolated anti-NMDAR encephalitis or a single demyelinating condition is essential, as overlapping forms may present with broader neurological involvement, different imaging findings, and possibly more complex therapeutic needs. Therefore, establishing evidence-based guidelines evidence-based guidelines [47] specifically designed for these intricate instances in order to enhance patient outcomes and reduce the possibility of long-term disability [9].

The increased frequency and high relapse rate (25%) among patients with demyelinating diseases [45] emphasize the importance of attentive observation [40] and sustained follow-up. The identification of predictive biomarkers and risk factors for relapse could help stratify patients and guide preventive strategies [38,52]. The favorable long-term prognosis observed in most patients with NMDAR + MOGAD overlap further supports the efficacy of aggressive immunotherapy, However, the higher relapse rates in overlapping syndromes compared to isolated disease forms underscore the importance of long-term monitoring and tailored prevention strategies [53].

Furthermore, the long-term consequences of these overlapping syndromes on cognitive function, quality of life, and overall prognosis warrant further investigation [41].

The co-occurrence of anti-NMDAR encephalitis with demyelinating diseases represents a significant diagnostic and therapeutic challenge. This systematic review underscores the need for a precision medicine approach that incorporates comprehensive immunological profiling to guide personalized treatment strategies and improve patient outcomes. While the growing body of literature has advanced our understanding of the complex interplay between anti-NMDAR encephalitis and demyelinating disorders, more research is needed to refine the pathogenic models, identify reliable biomarkers, and develop evidence-based management guidelines that address both the isolated and overlapping disease states.

## Conclusion

Overlapping syndromes of anti-NMDAR encephalitis and demyelinating diseases represent a complex clinical entity with distinct characteristics compared to isolated cases. These syndromes may feature milder encephalitis symptoms in some patients yet exhibit atypical clinical features, higher frequencies of MRI abnormalities, and variable therapeutic responses. Critically, the presence of MS, though rare, further expands the spectrum of possible overlaps. Early and accurate diagnosis supported by appropriate antibody testing methods and adherence to standardized criteriaand aggressive immunotherapy are crucial for improving outcomes and reducing the risk of relapse and long-term sequelae. Recognizing the differences between isolated and overlapping conditions will guide clinicians toward more tailored immunotherapies and long-term monitoring strategies, ultimately enhancing patient care and prognosis.

## Supplementary Material

Supplementary_1.docx

Supplementary_2.docx

## Data Availability

The data underpinning the results of this study can be obtained from the corresponding author upon a reasonable request.

## References

[1] Kovac S, Alferink J, Ahmetspahic D, et al. Update on anti-*N*-methyl-d-aspartate receptor encephalitis. Nervenarzt. 2018;89(1):99–112. doi: 10.1007/s00115-017-0405-0. 28932896

[2] Dalmau J, Lancaster E, Martinez-Hernandez E, et al. Clinical experience and laboratory investigations in patients with anti-NMDAR encephalitis. Lancet Neurol. 2011;10(1):63–74. doi: 10.1016/S1474-4422(10)70253-2. 21163445 PMC3158385

[3] Dalmau J, Armangué T, Planagumà J, et al. An update on anti-NMDA receptor encephalitis for neurologists and psychiatrists: mechanisms and models. Lancet Neurol. 2019;18(11):1045–1057. doi: 10.1016/S1474-4422(19)30244-3. 31326280

[4] Guasp M, Dalmau J. Encephalitis associated with antibodies against the NMDA receptor. Med Clin (Barc). 2018;151(2):71–79. doi: 10.1016/j.medcle.2018.05.020. 29183618

[5] Titulaer MJ, Höftberger R, Iizuka T, et al. Overlapping demyelinating syndromes and anti-*N*-methyl-d-aspartate receptor encephalitis. Ann Neurol. 2014;75(3):411–428. doi: 10.1002/ana.24117. 24700511 PMC4016175

[6] Nan D, Zhang Y, Han J, et al. Clinical features and management of coexisting anti-*N*-methyl-d-aspartate receptor encephalitis and myelin oligodendrocyte glycoprotein antibody-associated encephalomyelitis: a case report and review of the literature. Neurol Sci. 2021;42(3):847–855. doi: 10.1007/s10072-020-04942-0. 33409829

[7] Chen W, Li Q, Wang T, et al. Overlapping syndrome of anti-*N*-methyl-d-aspartate receptor encephalitis and anti-myelin oligodendrocyte glycoprotein inflammatory demyelinating diseases: a distinct clinical entity? Mult Scler Relat Disord. 2021;52:103020. doi: 10.1016/j.msard.2021.103020. 34034214

[8] Tao S, Zhang Y, Ye H, et al. AQP4-IgG-seropositive neuromyelitis optica spectrum disorder (NMOSD) coexisting with anti-*N*-methyl-d-aspartate receptor (NMDAR) encephalitis: a case report and literature review. Mult Scler Relat Disord. 2019;35:185–192. doi: 10.1016/j.msard.2019.07.008. 31398657

[9] Wingerchuk DM, Banwell B, Bennett JL, et al. International consensus diagnostic criteria for neuromyelitis optica spectrum disorders. Neurology. 2015;85(2):177–189. doi: 10.1212/WNL.0000000000001729. 26092914 PMC4515040

[10] Lennon VA, Wingerchuk DM, Kryzer TJ, et al. A serum autoantibody marker of neuromyelitis optica: distinction from multiple sclerosis. Lancet. 2004;364(9451):2106–2112. doi: 10.1016/S0140-6736(04)17551-X. 15589308

[11] Compston A, Coles A. Multiple sclerosis. Lancet. 2008;372(9648):1502–1517. doi: 10.1016/S0140-6736(08)61620-7. 18970977

[12] Etemadifar M, Fereidan-Esfahani M, Sedaghat N, et al. Non-infectious meningitis and CNS demyelinating diseases: a conceptual review. Rev Neurol (Paris). 2023;179(6):533–547. doi: 10.1016/j.neurol.2022.10.006. 36781321

[13] Zhang S, Yang Y, Liu W, et al. Clinical characteristics of anti-*N*-methyl-d-aspartate receptor encephalitis overlapping with demyelinating diseases: a review. Front Immunol. 2022;13:857443. doi: 10.3389/fimmu.2022.857443. 35837405 PMC9273846

[14] Molazadeh N, Bose G, Lotan I, et al. Autoimmune diseases and cancers overlapping with myelin oligodendrocyte glycoprotein antibody-associated disease (MOGAD): a systematic review. Mult Scler J Exp Transl Clin. 2022;8(4):20552173221128170. doi: 10.1177/20552173221128170. 36311694 PMC9597055

[15] Dalmau J, Geis C, Graus F. Autoantibodies to synaptic receptors and neuronal cell surface proteins in autoimmune diseases of the central nervous system. Physiol Rev. 2017;97(2):839–887. doi: 10.1152/physrev.00010.2016. 28298428 PMC5539405

[16] Planagumà J, Leypoldt F, Mannara F, et al. Human *N*-methyl d-aspartate receptor antibodies alter memory and behaviour in mice. Brain. 2015;138(Pt 1):94–109. doi: 10.1093/brain/awu310. 25392198 PMC4285189

[17] Tobin WO, Pittock SJ. Autoimmune neurology of the central nervous system. Continuum (Minneap Minn). 2017;23(3, Neurology of Systemic Disease):627–653. doi: 10.1212/CON.0000000000000487. 28570322

[18] Hacohen Y, Deiva K, Pettingill P, et al. *N*-methyl-d-aspartate receptor antibodies in post-herpes simplex virus encephalitis neurological relapse. Mov Disord. 2014;29(1):90–96. doi: 10.1002/mds.25626. 24014096

[19] Gong S, Zhang WH, Ren HT, et al. [Clinical observation on the overlapping syndrome of myelin oligodendrocyte glycoprotein antibody and anti-*N*-methyl-d aspartate receptor in children]. Zhonghua Er Ke Za Zhi. 2020;58(7):581–585. doi: 10.3760/cma.j.cn112140-20191209-00788. 32605343

[20] Waschbisch A, Kallmünzer B, Schwab S, et al. Demyelinating disease and anti-*N*-methyl-d-aspartate receptor immunoglobulin G antibodies: a case report. BMC Res Notes. 2014;7(1):948. doi: 10.1186/1756-0500-7-948. 25539977 PMC4307172

[21] Ren B-Y, Guo Y, Han J, et al. Case report: anti-NMDAR encephalitis with anti-MOG CNS demyelination after recurrent CNS demyelination. Front Neurol. 2021;12:639265. doi: 10.3389/fneur.2021.639265. 33716942 PMC7943444

[22] Kim HW, Lamb C, Jamali S, et al. Mystery case: anti-NMDAR encephalitis with overlapping demyelinating syndrome. Neurology. 2020;94(17):e1866–e1869. doi: 10.1212/WNL.0000000000009320. 32234822

[23] Seyed Ahadi M, Ghadiri F, Sahraian MA, et al. Anti-*N*-methyl-d-aspartate receptor encephalitis in a patient with multiple sclerosis on dimethyl fumarate: a case report. Neurol Sci. 2021;42(9):3929–3931. doi: 10.1007/s10072-021-05385-x. 34155564

[24] Weiss D, Kertzscher L, Degering M, et al. Anti-NMDA receptor encephalitis and overlapping demyelinating disorder in a 20-year old female with borderline personality disorder: proposal of a diagnostic and therapeutic algorithm for autoimmune encephalitis in psychiatric patients "case report". BMC Psychiatry. 2021;21(1):355. doi: 10.1186/s12888-021-03269-0. 34266413 PMC8280600

[25] Fan S, Xu Y, Ren H, et al. Comparison of myelin oligodendrocyte glycoprotein (MOG)-antibody disease and AQP4-IgG-positive neuromyelitis optica spectrum disorder (NMOSD) when they co-exist with anti-NMDA (*N*-methyl-d-aspartate) receptor encephalitis. Mult Scler Relat Disord. 2018;20:144–152. doi: 10.1016/j.msard.2018.01.007. 29414288

[26] Cherian A, Divya KP, Shetty SC, et al. Coexistent MOG, NMDAR, CASPR2 antibody positivity: triumph over the triumvirate. Mult Scler Relat Disord. 2020;46:102468. doi: 10.1016/j.msard.2020.102468. 32906000

[27] Du L, Wang H, Zhou H, et al. Anti-NMDA receptor encephalitis concomitant with myelin oligodendrocyte glycoprotein antibody diseases: a retrospective observational study. Medicine (Baltimore). 2020;99(31):e21238. doi: 10.1097/MD.0000000000021238. 32756102 PMC7402765

[28] Xu X, Lu Q, Huang Y, et al. Anti-NMDAR encephalitis: a single-center, longitudinal study in China. Neurol Neuroimmunol Neuroinflamm. 2020;7(1):1–9. doi: 10.1212/NXI.0000000000000633. PMC6857906 31619447

[29] Jarius S, Ruprecht K, Kleiter I, et al. MOG-IgG in NMO and related disorders: a multicenter study of 50 patients. Part 2: epidemiology, clinical presentation, radiological and laboratory features, treatment responses, and long-term outcome. J Neuroinflamm. 2016;13(1):280. doi: 10.1186/s12974-016-0718-0. PMC5086042 27793206

[30] Sarigecili E, Cobanogullari MD, Komur M, et al. A rare concurrence: antibodies against myelin oligodendrocyte glycoprotein and *N*-methyl-d-aspartate receptor in a child. Mult Scler Relat Disord. 2019;28:101–103. doi: 10.1016/j.msard.2018.12.017. 30590238

[31] Ren Y, Chen X, He Q, et al. Co-occurrence of anti-*N*-methyl-d-aspartate receptor encephalitis and anti-myelin oligodendrocyte glycoprotein inflammatory demyelinating diseases: a clinical phenomenon to be taken seriously. Front Neurol. 2019;10:1271. doi: 10.3389/fneur.2019.01271. 31866928 PMC6904358

[32] Sinani AA, Maawali SA, Alshekaili J, et al. Overlapping demyelinating syndrome (Neuromyelitis optica spectrum disorders NMOSD with anti-NMDA receptor encephalitis): a case report. Mult Scler Relat Disord. 2020;42:102153. doi: 10.1016/j.msard.2020.102153. 32413838

[33] Li Y, Luo H, Zheng Y, et al. Pediatric anti-NMDAR encephalitis with demyelination on brain MRI: a single center study. Mult Scler Relat Disord. 2023;80:105063. doi: 10.1016/j.msard.2023.105063. 37913674

[34] Kang Q, Kang H, Liu S, et al. Clinical characteristics of Chinese pediatric patients positive for anti-NMDAR and MOG antibodies: a case series. Front Neurol. 2023;14:1279211. doi: 10.3389/fneur.2023.1279211. 38249740 PMC10796507

[35] Yang J-X, Yang M-M, Han Y-J, et al. FLAIR-hyperintense lesions in anti-MOG-associated encephalitis with seizures overlaying anti-N-methyl-D-aspartate receptor encephalitis: a case report and literature review. Front Immunol. 2023;14:1149987. doi: 10.3389/fimmu.2023.1149987. 37138864 PMC10150000

[36] Du B-Q, Lai Q-L, Li E-C, et al. Myelin oligodendrocyte glycoprotein antibody and *N*-methyl-d-aspartate receptor antibody overlapping syndrome: insights from the recent case reports. Clin Exp Immunol. 2024;215(1):27–36. doi: 10.1093/cei/uxad109. 37724585 PMC10776248

[37] Dai Y, Yuan Y, Bi F, et al. Clinical features of adult patients with positive NMDAR-IgG coexisting with MOG-IgG. Neurol Sci. 2024;45(9):4481–4492. doi: 10.1007/s10072-024-07474-z. 38523205

[38] Tao R, Qin C, Chen M, et al. Unilateral cerebral cortical encephalitis with epilepsy: a possible special phenotype of MOG antibody-associated disorders. Int J Neurosci. 2020;130(11):1161–1165. doi: 10.1080/00207454.2020.1720676. 31971044

[39] Hamid SHM, Whittam D, Mutch K, et al. What proportion of AQP4-IgG-negative NMO spectrum disorder patients are MOG-IgG positive? A cross sectional study of 132 patients. J Neurol. 2017;264(10):2088–2094. doi: 10.1007/s00415-017-8596-7. 28840314 PMC5617862

[40] Gresa-Arribas N, Titulaer MJ, Torrents A, et al. Antibody titres at diagnosis and during follow-up of anti-NMDA receptor encephalitis: a retrospective study. Lancet Neurol. 2014;13(2):167–177. doi: 10.1016/S1474-4422(13)70282-5. 24360484 PMC4006368

[41] de Montmollin E, Demeret S, Brulé N, et al. Anti-*N*-methyl-d-aspartate receptor encephalitis in adult patients requiring intensive care. Am J Respir Crit Care Med. 2017;195(4):491–499. doi: 10.1164/rccm.201603-0507OC. 27552490

[42] Fujihara K. Neuromyelitis optica spectrum disorders: still evolving and broadening. Curr Opin Neurol. 2019;32(3):385–394. doi: 10.1097/WCO.0000000000000694. 30893099 PMC6522202

[43] Dos Passos GR, Oliveira LM, da Costa BK, et al. MOG-IgG-associated optic neuritis, encephalitis, and myelitis: lessons learned from neuromyelitis optica spectrum disorder. Front Neurol. 2018;9:217. doi: 10.3389/fneur.2018.00217. 29670575 PMC5893792

[44] Liu P, Yan H, Li H, et al. Overlapping anti-NMDAR encephalitis and multiple sclerosis: a case report and literature review. Front Immunol. 2023;14:1088801. doi: 10.3389/fimmu.2023.1088801. 36793718 PMC9923169

[45] Titulaer MJ, McCracken L, Gabilondo I, et al. Treatment and prognostic factors for long-term outcome in patients with anti-NMDA receptor encephalitis: an observational cohort study. Lancet Neurol. 2013;12(2):157–165. doi: 10.1016/S1474-4422(12)70310-1. 23290630 PMC3563251

[46] Armangue T, Spatola M, Vlagea A, et al. Frequency, symptoms, risk factors, and outcomes of autoimmune encephalitis after herpes simplex encephalitis: a prospective observational study and retrospective analysis. Lancet Neurol. 2018;17(9):760–772. doi: 10.1016/S1474-4422(18)30244-8. 30049614 PMC6128696

[47] Cobo-Calvo A, Ruiz A, Maillart E, et al. Clinical spectrum and prognostic value of CNS MOG autoimmunity in adults: the MOGADOR study. Neurology. 2018;90(21):e1858–e1869. doi: 10.1212/WNL.0000000000005560. 29695592

[48] Gastaldi M, Scaranzin S, Jarius S, et al. Cell-based assays for the detection of MOG antibodies: a comparative study. J Neurol. 2020;267(12):3555–3564. doi: 10.1007/s00415-020-10024-0. 32623596

[49] Alkabie S, Budhram A. Testing for antibodies against aquaporin-4 and myelin oligodendrocyte glycoprotein in the diagnosis of patients with suspected autoimmune myelopathy. Front Neurol. 2022;13:912050. doi: 10.3389/fneur.2022.912050. 35669883 PMC9163833

[50] Gilligan M, McGuigan C, McKeon A. Autoimmune central nervous system disorders: antibody testing and its clinical utility. Clin Biochem. 2024;126:110746. doi: 10.1016/j.clinbiochem.2024.110746. 38462203 PMC11016295

[51] Hacohen Y, Wong YY, Lechner C, et al. Disease course and treatment responses in children with relapsing myelin oligodendrocyte glycoprotein antibody-associated disease. JAMA Neurol. 2018;75(4):478–487. doi: 10.1001/jamaneurol.2017.4601. 29305608 PMC5885190

[52] Ogawa R, Nakashima I, Takahashi T, et al. MOG antibody-positive, benign, unilateral, cerebral cortical encephalitis with epilepsy. Neurol Neuroimmunol Neuroinflamm. 2017;4(2):e322. doi: 10.1212/NXI.0000000000000322. 28105459 PMC5241006

[53] Camellino D, Matteson EL, Buttgereit F, et al. Monitoring and long-term management of giant cell arteritis and polymyalgia rheumatica. Nat Rev Rheumatol. 2020;16(9):481–495. doi: 10.1038/s41584-020-0458-5. 32759996

