# Supplementary material for: The spectrum of overlapping anti-NMDAR encephalitis and demyelinating syndromes: a systematic review of presentation, diagnosis, management, and outcomes
Source: Ann Med. 2025 Jul 3;57(1):2517813. doi: 10.1080/07853890.2025.2517813 (PMC12231328; doi:10.1080/07853890.2025.2517813)
Supplement: Supplementary_1.docx [file IANN_A_2517813_SM1347.docx]

Supplementary File 2: Search Strategy and Methodology

1. Information Sources

The following electronic databases were systematically searched:

- MEDLINE (via PubMed)

- Embase

- Scopus

- Web of Science

- Cochrane Library

- Google Scholar

2. Search Period

All databases were searched from their inception to March 2024.

3. Search Strategy

3.1 MeSH terms and free-text keywords:

Concept 1: Anti-NMDAR Encephalitis

"anti-N-methyl-D-aspartate receptor encephalitis" OR "NMDAR encephalitis" OR "anti-NMDAR encephalitis"

Concept 2: Demyelinating Diseases

"myelin oligodendrocyte glycoprotein" OR "MOG" OR "MOGAD" OR "neuromyelitis optica spectrum disorder" OR "NMOSD" OR "multiple sclerosis" OR "MS" OR "demyelinating disease"

3.2 Combined search:

(Concept 1) AND (Concept 2)

1. **Sample Search String**

**PubMed**

((("anti-N-methyl-D-aspartate receptor encephalitis"[MeSH Terms] OR "NMDAR encephalitis"[Title/Abstract] OR "anti-NMDAR encephalitis"[Title/Abstract])) AND ("myelin oligodendrocyte glycoprotein"[MeSH Terms] OR "MOG"[Title/Abstract] OR "MOGAD"[Title/Abstract] OR "neuromyelitis optica spectrum disorder"[MeSH Terms] OR "NMOSD"[Title/Abstract] OR "multiple sclerosis"[MeSH Terms] OR "MS"[Title/Abstract] OR "demyelinating disease"[MeSH Terms]))

**Scopus**

(TITLE-ABS-KEY("anti-N-methyl-D-aspartate receptor encephalitis" OR "NMDAR encephalitis" OR "anti-NMDAR encephalitis")) AND (TITLE-ABS-KEY("myelin oligodendrocyte glycoprotein" OR "MOG" OR "MOGAD" OR "neuromyelitis optica spectrum disorder" OR "NMOSD" OR "multiple sclerosis" OR "MS" OR "demyelinating disease"))

**Web of Science**

TS=("anti-N-methyl-D-aspartate receptor encephalitis" OR "NMDAR encephalitis" OR "anti-NMDAR encephalitis") AND TS=("myelin oligodendrocyte glycoprotein" OR "MOG" OR "MOGAD" OR "neuromyelitis optica spectrum disorder" OR "NMOSD" OR "multiple sclerosis" OR "MS" OR "demyelinating disease")

**Cochrane Library**

("anti-N-methyl-D-aspartate receptor encephalitis" OR "NMDAR encephalitis" OR "anti-NMDAR encephalitis") AND ("myelin oligodendrocyte glycoprotein" OR "MOG" OR "MOGAD" OR "neuromyelitis optica spectrum disorder" OR "NMOSD" OR "multiple sclerosis" OR "MS" OR "demyelinating disease")

**Google Scholar**

"anti-N-methyl-D-aspartate receptor encephalitis" OR "NMDAR encephalitis" OR "anti-NMDAR encephalitis" AND "myelin oligodendrocyte glycoprotein" OR "MOG" OR "MOGAD" OR "neuromyelitis optica spectrum disorder" OR "NMOSD" OR "multiple sclerosis" OR "MS" OR "demyelinating disease"

5. Search Limits

- Language: English

- Study designs: Observational studies, cohort studies, case-control studies, and case series

- Species: Humans

6. Supplementary Search Methods

To ensure comprehensive coverage, the following additional search methods were employed:

- Manual review of reference lists from included studies

- Forward citation tracking of key papers

- Consultation with subject matter experts to identify potentially overlooked relevant studies
